# A phylogenomic analysis of *Limosilactobacillus reuteri* reveals ancient and stable evolutionary relationships with rodents and birds and zoonotic transmission to humans

**DOI:** 10.1186/s12915-023-01541-1

**Published:** 2023-03-13

**Authors:** Fuyong Li, Xudong Li, Christopher C. Cheng, Dalimil Bujdoš, Stephanie Tollenaar, David J. Simpson, Guergana Tasseva, Maria Elisa Perez-Muñoz, Steven Frese, Michael G. Gänzle, Jens Walter, Jinshui Zheng

**Affiliations:** 1 Department of Agricultural, Food and Nutritional Science, University of Alberta, Edmonton, AB T6G 2E1 Canada; 2 Department of Infectious Diseases and Public Health, Jockey Club College of Veterinary Medicine and Life Sciences, City University of Hong Kong, Kowloon, Hong Kong SAR China; 3 State Key Laboratory of Agricultural Microbiology, Huazhong Agricultural University, Wuhan, 430070 China; 4 Hubei Key Laboratory of Agricultural Bioinformatics, Huazhong Agricultural University, Wuhan, 430070 China; 5 Department of Biological Sciences, University of Alberta, Edmonton, AB T6G 2E1 Canada; 6 School of Microbiology, and Department of Medicine, APC Microbiome Ireland, University College Cork, Cork, T12 YT20 Ireland; 7 Department of Nutrition, University of Nevada, Reno, NV 89557 USA

**Keywords:** *Limosilactobacillus reuteri*, Phylogenomics, Lifestyle, Evolution, Epithelial colonization

## Abstract

**Background:**

Gut microbes play crucial roles in the development and health of their animal hosts. However, the evolutionary relationships of gut microbes with vertebrate hosts, and the consequences that arise for the ecology and lifestyle of the microbes are still insufficiently understood. Specifically, the mechanisms by which strain-level diversity evolved, the degree by which lineages remain stably associated with hosts, and how their evolutionary history influences their ecological performance remain a critical gap in our understanding of vertebrate-microbe symbiosis.

**Results:**

This study presents the characterization of an extended collection of strains of *Limosilactobacillus reuteri* and closely related species from a wide variety of hosts by phylogenomic and comparative genomic analyses combined with colonization experiments in mice to gain insight into the long-term evolutionary relationship of a bacterial symbiont with vertebrates. The phylogenetic analysis of *L. reuteri* revealed early-branching lineages that primarily consist of isolates from rodents (four lineages) and birds (one lineage), while lineages dominated by strains from herbivores, humans, pigs, and primates arose more recently and were less host specific. Strains from rodent lineages, despite their phylogenetic divergence, showed tight clustering in gene-content-based analyses. These *L. reuteri* strains but not those ones from non-rodent lineages efficiently colonize the forestomach epithelium of germ-free mice. The findings support a long-term evolutionary relationships of *L. reuteri* lineages with rodents and a stable host switch to birds. Associations of *L. reuteri* with other host species are likely more dynamic and transient. Interestingly, human isolates of *L. reuteri* cluster phylogenetically closely with strains from domesticated animals, such as chickens and herbivores, suggesting zoonotic transmissions.

**Conclusions:**

Overall, this study demonstrates that the evolutionary relationship of a vertebrate gut symbiont can be stable in particular hosts over time scales that allow major adaptations and specialization, but also emphasizes the diversity of symbiont lifestyles even within a single bacterial species. For *L. reuteri*, symbiont lifestyles ranged from autochthonous, likely based on vertical transmission and stably aligned to rodents and birds over evolutionary time, to allochthonous possibly reliant on zoonotic transmission in humans. Such information contributes to our ability to use these microbes in microbial-based therapeutics.

**Supplementary Information:**

The online version contains supplementary material available at 10.1186/s12915-023-01541-1.

## Background

Vertebrates, including humans, are colonized by complex microbial communities central to host physiology. The evolution of these communities and individual microbes within them provides information to understand the characteristic of this symbiosis. The taxonomic profiles of vertebrate microbiota often cluster by host [1], and in rare cases, the phylogeny of host and the phylogeny of microbial symbionts appeared congruent [2]. This phenomenon has been referred to as phylosymbiosis [3] and was interpreted by some as evidence for the existence of a “hologenome,” meaning that a subset of the microbial community, as well as the microbial genes, show stable transmission over evolutionary times and strict inheritance [4]. Stable transmission has recently been supported by findings from Suzuki et al. [5], who found that dozens of bacterial lineages showed evidence of co-diversification with human populations. However, the hologenome concept has been criticized for being too restrictive [6] and for not accounting for the complexity of host-microbe evolutionary relationships [7]. In this context, it is crucial to consider that the phylogenetic clustering of whole microbiomes can arise through both evolutionary and ecological processes. The extent of co-diversification within the gut microbiota has remained contentious [8], and the phylogenetic patterns of co-speciation that have been detected for gut symbionts of hominids [9] were not confirmed in studies that analyzed a larger sample size [10]. Accordingly, the authors of the latter study urged caution that host-restricted bacterial clades can arise through ecological selection, vicariance, or drift rather than co-speciation.

A limitation of most studies that tested the hologenome and co-speciation hypotheses is that they relied on genomic data (mostly sequence analyses of marker genes), and therefore cannot reveal relevant functional traits, microbial adaptations, and the underlying genetic mechanisms of the evolutionary process. The functional consequences in terms of ecological fitness and evolutionary trade-offs in relation to the microbial colonization of particular hosts have not been assessed using appropriate model systems. How microbial symbionts evolve with their vertebrate hosts therefore remains poorly understood. Complementing taxonomic or metagenomics analyses with phylogenomic analyses of bacterial species is amenable to functional experiments in animal models, which can provide more robust insights into the evolution and host adaptation of bacteria [11]. These tools have been extensively used to investigate the evolution of host-adapted pathogens [12] and test for stable associations with hosts and host switches [13, 14], but have rarely been used to elucidate the evolution of bacterial gut symbionts.

*Limosilactobacillus reuteri* has been established as a model for the study of the evolution of a vertebrate host-associated bacterial symbiont. This species diversified into phylogenetic lineages that are remarkably host specific [15–18] and were recently described as subspecies [19]. The phylogenetic lineages possess a specific gene content that reflect niche characteristics of particular hosts [16, 17]. These phylogenetic analyses have been combined with colonization experiments in gnotobiotic animals and even humans [16, 18], allowing functional studies that are unavailable for other microbial symbionts. This research has established host adaptation of *L. reuteri* lineages in mice and chickens [16, 18], identified the major mechanisms for host specificity in mice [17], and functionally characterized genes and metabolic traits that contributed to ecological fitness in rodents [16, 17, 20–22]. To our knowledge, *L. reuteri* is the only vertebrate gut microbe for which a stable and host-specific evolutionary relationship has been experimentally established [23], providing unique insight into the lifestyle and ecological strategies of a bacterial symbiont in relation to its vertebrate hosts as well as the mechanisms by which such associations are maintained.

Our understanding, however, of the evolutionary history of *L. reuteri* is incomplete. First, the evolutionary origin of *L. reuteri* and the evolutionary processes that allowed this species to diversify into a range of host species are insufficiently understood. Second, while *L. reuteri* seems to maintain evolutionary stable relationships with rodents and poultry [17, 18], several lineages include strains from other hosts, e.g., humans, swine, or herbivores [18, 24], posing questions about the exact ecological strategy of strains within these lineages. Third, current knowledge is confounded by sampling bias, as most isolates that have been obtained so far originate from humans and domesticated or laboratory animals. Therefore, the robustness of host associations in phylogenetic lineages and the boundaries of host specialization remain unclear.

This study therefore aimed to provide a phylogenomic analysis of 94 newly isolated strains of *L. reuteri* isolated from primates (including humans), rodents, birds, and pigs, including isolates from both wild and zoo animals, together with 88 *L. reuteri* genome sequences that are available in public databases, and 25 genomes of closely related species [19]. Core-genome phylogeny, analysis of the gene content of strains of *L. reuteri* from different hosts or lineages, the exchange of phage genes, and the evolutionary history of host switches were analyzed to elucidate the mechanisms by which *L. reuteri* lineages evolve. To assess the functional consequence of evolution, we determined colonization of the forestomach epithelium in mono-associated mice, which was established in previous studies to be highly specific to strains of rodent origin [16, 17].

## Results

### Phylogenomic analysis of an expanded strain collection of *L. reuteri*

We expanded our strain collection of *L. reuteri* [15, 16, 18] through isolating from fecal and gut samples of zoo and wild animals, including birds (6 species), non-human primates (9 species), wild rodents (8 species), and humans from both industrialized and non-industrialized societies (Additional file 1: Table S1). This strain collection represents a greater depth of host phylogenetic diversity to enable the analysis of the genetic variation and host specificity of *L*.* reuteri* strains. We also attempted to cultivate *L*. *reuteri* from fecal samples of pig species from the wild and zoos (e.g., peccaries, Vietnamese pot-bellied, red river hog, and wild boar), but these attempts were not successful (data not shown). We rarefied the strain collection by selecting only one isolate per individual host and sequenced their 94 genomes. Together with published genomes of *L*. *reuteri* strains with reliable host assignments, we generated a dataset of a total of 182 genomes from more than 40 different vertebrate host species (Additional file 1: Table S1).

**Fig. 1.**
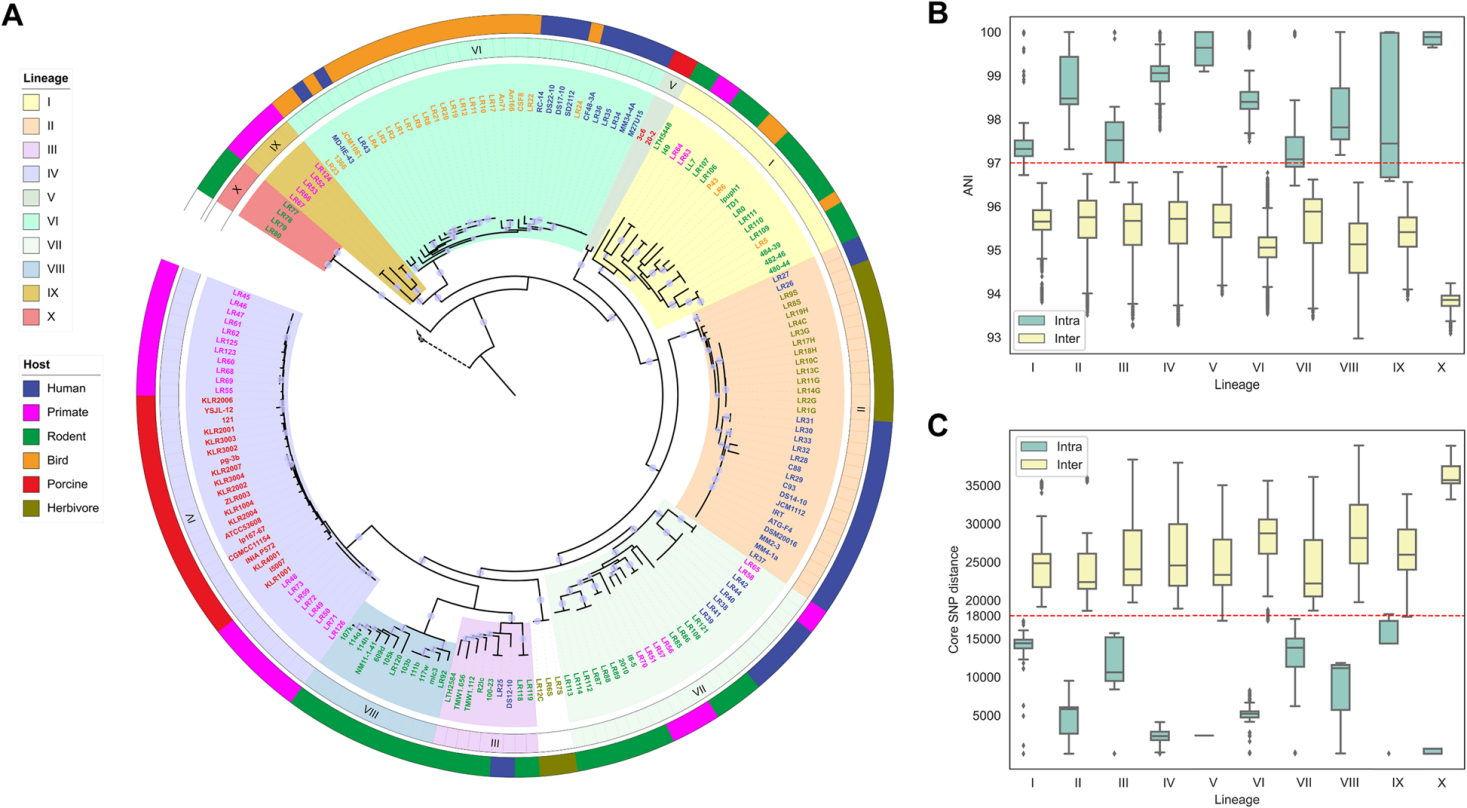
Population structure of *Limosilactobacillus reuteri*.** A** Maximum likelihood tree of 182 *L. reuteri* strains based on the core-genome alignment, excluding recombination events. The tree was rooted with *L. agrestis*, which has a close phylogenetic relationship with *L. reuteri* [19]. Bootstrap values above 90% are marked as gray dots on the branches. The ten lineages were defined with the population structure analysis using BAPS [25]. Label colors refer to different vertebrate hosts, green for rodent, red for porcine, blue for human, pink for primate except human, yellow for bird, and olive for herbivore. **B** ANI plot of genome sequence pairs belonging to the same or different lineages. **C** SNP distance of core-genome pairs. SNP-dists (https://github.com/tseemann/snp-dists) was used to extracted SNP distance from the core-genome alignment that was obtained through Roary [26]. The red dotted lines in **B** and **C** separate a majority of intra-lineage values from inter-lineage values

A phylogenetic tree based on 490 core genes that were not affected by recombination events revealed that strains of *L. reuteri* clustered in ten phylogenetic lineages, supported by the bootstrap value higher than 90% for each lineage (Fig. 1A). The genetic relationship of lineages was interrogated by calculation of the pairwise average nucleotide identity (ANI; Fig. 1B; Additional file 2: Table S2), core-gene single-nucleotide polymorphism (SNP) distance (Fig. 1C), gene content distance (Additional file 4: Fig. S1), and genome size (Additional file 4: Fig. S2). All these metrics support the evolution of *L. reuteri* into distinct lineages. Six of the ten lineages conform to previously identified lineages [15, 16, 18], which were recently described as subspecies of *L. reuteri* subsp. *murium, reuteri*, *rodentium*, *suis*, *porcinus*, and *kinnaridis* [19]. We detected four novel phylogenetic lineages represented by four or more strains (lineages VII—X). Three strains from herbivores [24] remain unassigned to any lineage (Fig. 1A).

**Fig. 2.**
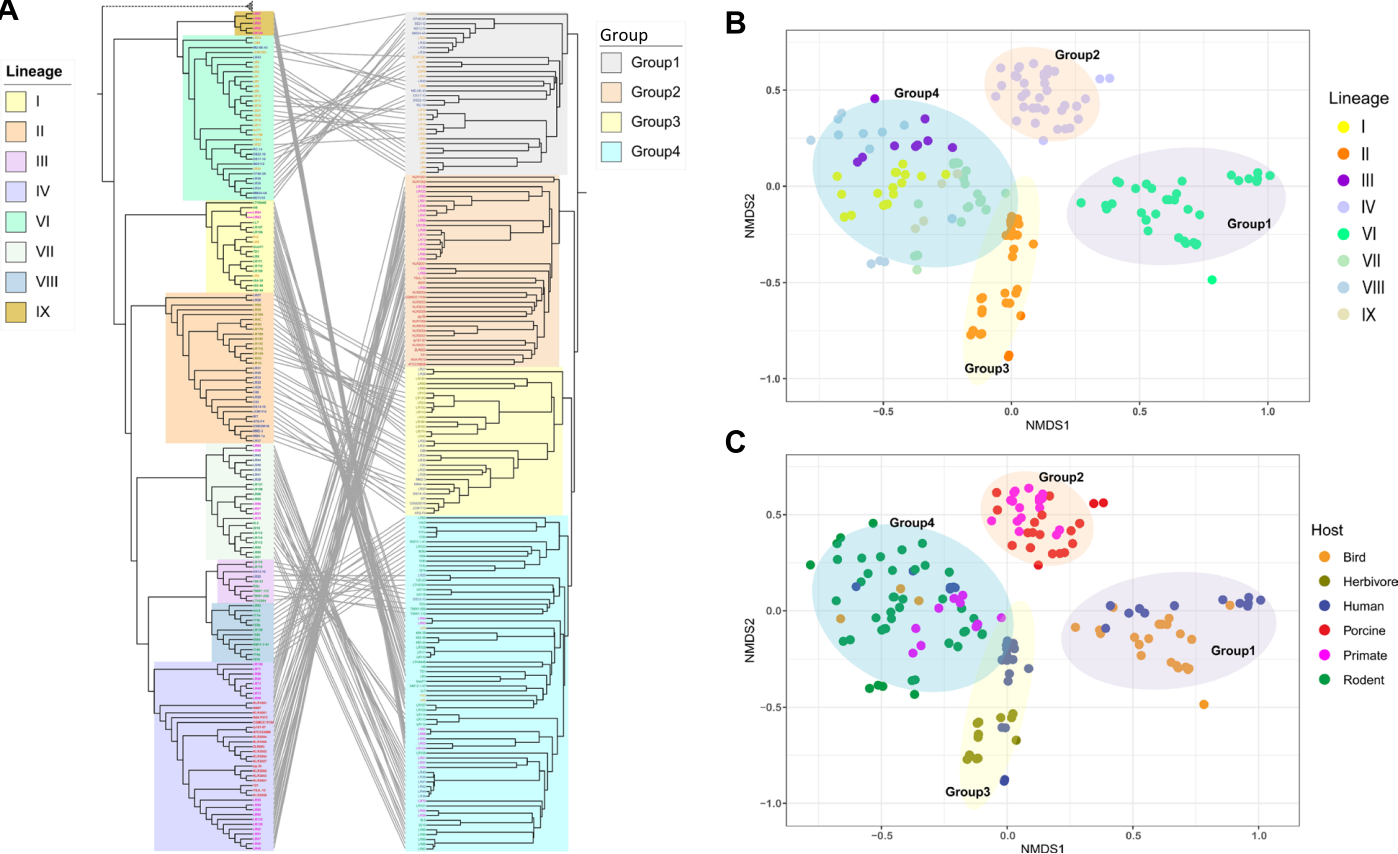
Gene content diversity of different lineages.** A** Juxtaposition of the topologies of the core-genome tree (left) and gene content tree (right). The gene content tree based on Jaccard distance matrix was built by the software MEGA-X using UPGMA method [27]. Core-genome tree was built as described for Fig. 1A. Nonmetric multidimensional scaling (NMDS) ordination based on Jaccard distance of each genome pairs colored by lineages (**B**) and hosts (**C**). Four Gene Content Groups were separated based on the Jaccard distance matrix. Ellipses denote 90% confidence intervals. The metaMDS function in the vegan package (https://github.com/vegandevs/vegan) was used to perform NMDS with the stress value of 0.2, and ggplot2 package (https://ggplot2.tidyverse.org) was applied for visualization. Gene Content Group 1 includes lineage VI which comprises isolates from birds and humans, Gene Content Group 2 includes lineage IV which comprises isolates from swine and primates, Gene Content Group 3 includes lineages II which comprises isolates from swine and humans, Gene Count Group 4 includes lineages I, III, VII, VIII, and IX which comprise isolates from rodents, humans, birds, and primates

Lineages I, III, VIII, and X contained exclusively or predominantly rodent isolates (Fig. 1A). Lineage VIII included strains that were previously included in lineage III based on a phylogenetic analysis of marker genes [15], but the higher resolution of genome-wide core-gene-based phylogeny, pairwise ANI, the distance of core-gene SNPs, and gene content support the existence of a distinct lineage (Figs. 1 and 2; Additional file 4: Fig. S1 & S2). Strains in lineages I, III, VIII, and X were isolated predominantly from hosts in the *Muridae*, true mice including field mice, and rats (Additional file 1: Table S1). Rodent isolates also comprised a sizable proportion of strains in lineage VII; these isolates were not obtained from *Muridae* but from diverse families in the order *Rodentia*. Lineage VII additionally included isolates from primates living in zoos and humans living in non-industrialized societies (Papua New Guinea). Lineages dominated by rodent strains displayed greater genetic diversities based on their lower intra-lineage ANI values and larger intra-lineage distance of core-gene SNPs compared to lineages IV, II, and VI (Figs. 1B, C).

As described previously [15, 18, 19, 28], lineage VI was composed of poultry and human isolates, and the addition of 19 new isolates from eight additional bird species supported the specificity of this lineage to birds (Fig. 1). The inclusion of newly available genomes from herbivore isolates [24] revealed that human strains of linage II [15] form a joint lineage with strains from herbivores. Swine isolates were all assigned to lineages IV and V [15, 18]. Most primate isolates clustered with porcine isolates in lineage IV, and all strains assigned to lineage IX were isolated from non-human primates (Fig. 1A).

### Analysis of *L. reuteri* based on gene content

The pan-genomes of all lineages remain open (Additional file 4: Fig. S3A), indicating the continued acquisition of genes that may contribute to adaptation to various hosts. To compare the gene content of lineages represented by no less than five strains, we calculated the Jaccard distance (JD) for each of genome pairs and constructed a gene content tree based on the JD matrix (Fig. 2A). Except of small differences (lineage IX was embraced in lineage VII), gene content analysis confirmed the existence of the phylogenetic lineages identified based on the core-gene-based phylogeny. However, the overall topology of the gene content tree changed, which clustered all rodent-associated lineages in the same clade (Fig. 2A). Bird isolates also clustered together (upper part of Fig. 2A).

Four different Gene Content Croups were clearly separated according to the gene content tree (Fig. 2A), nonmetric multidimensional scaling (NMDS) ordination (Fig. 2B, C), and analysis of similarities (ANOSIM; *p* = 0.001, *R* = 0.857; Additional file 4: Fig. S4). Gene Content Groups 1, 2, and 3 matched with three non-rodent phylogenetic lineages VI (bird and human), IV (pig and non-human primate), and II (humans and herbivores), respectively, while all the rodent lineages were grouped as Gene Content Group 4 (rodent-associated lineages I, III, VII, VIII, and the primate-associated lineage IX) (Fig. 2). Taken together, the clear gene content clustering based on the host origin of isolates indicates that the phylogenetically diverse lineages primarily associated with rodents share a common set of genes and are functionally equivalent.

### Functional analysis of Gene-Content-Group-specific genes

**Fig. 3.**
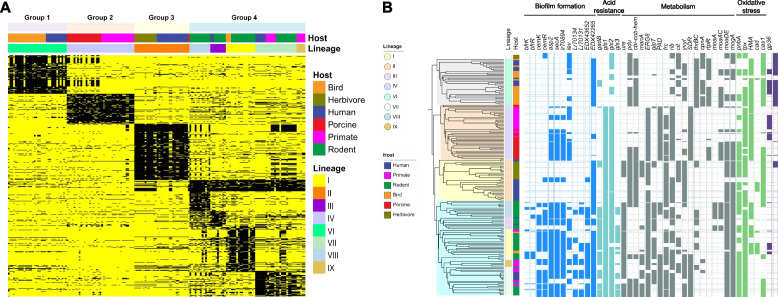
Distribution of host- and lineage-specific genes.** A** Presence and absence of gene families specific to different lineages or hosts (yellow, present; black, absent). Lineage- and host-specific genes were analyzed using Scoary [33]. Gene families with sensitivity and specificity both above 70% and Bonferroni-adjusted *p*-value less than 0.05 were included. **B** Distribution of key functional gene clusters and genes among different lineages. Five categories of genes are shown in different colors, from left to right: biofilm formation (blue), acid resistance (turquoise), metabolism (gray), oxidative stress (green), and CRISPR-Cas and phage (purple). The function of the genes are described as follows: *bfrKR*, two-component regulatory system; *cemKR*, two-component regulatory system; *asp2*, accessory Sec system protein Asp2; *secA*, protein translocase subunit SecA2; *Lr70894*, *secA2* operon component; *lev*, levansucrase; *Lr70134*, *Lr70131*, *EDX43652*, *EDX42265*, large surface proteins (Lsp) containing LPXTG cell wall anchoring motif; *gadB*, glutamate decarboxylase; *gls1*, *gls2*, *gls3*, the 3 glutaminases; *ure*, urease gene cluster for urea metabolism; *pdu* and *cbi*, part of the *pdu*-*cbi-cob-hem* gene cluster that mediates 1,2-propanediol and glycerol utilization, and cobalamin biosynthesis; *manA*, mannose-6-phosphate isomerase; *ERG8*, phosphomevalonate kinase; *glpT*, glycerol-3-phosphate transporter; *PAD*, phenolic acid decarboxylase; *frc*, formyl-coenzyme A transferase; *rib*, gene cluster for riboflavin biosynthesis; *cit*, citrate lyase cluster; *yvyI*, mannose-6-phosphate isomerase YvyI; *SDR*, short-chain dehydrogenases/reductases; *thrBC*, threonine biosynthetic cluster; *cpnA*, cyclopentanol dehydrogenase; *rtpR*, adenosylcobalamin-dependent ribonucleoside-triphosphate reductase; *moaA*/*moaAC*/*moaDE*/*mogA*/*mobAB*, genes for molybdenum cofactor (Moco) biosynthesis; *nreB*, oxygen sensor histidine kinase NreB; *pnbA*, nitroreductase-like family protein; *tpx*, redoxin domain protein; *HMA*, heavy metal translocating P-type ATPase; *cad*, cadmium resistance transporter; *cas1*, CRISPR-Cas system; *gp36*, phage phi-C31 major capsid gp36-like protein

To further understand the role of Gene-Content-Group-specific genes in the evolutionary adaptations of *L. reuteri*, we performed pan-genome-wide association analyses (Pan-GWAS) based on Gene Content Groups and phylogenetic lineages, respectively. We demonstrated that each Gene Content Group harbored a set of specific accessory genes (Fig. 3A; Additional file 3: Table S3), and each lineage within Gene Content Group 4 also contained a considerable number of lineage-specific genes. Several of the specific genes of Genome Content Group 4 were previously identified in rodent strains to contribute to biofilm formation and/or ecological fitness in the rodent intestine [16, 17]. For example, genes contributing to acid resistance and biofilm formation in vitro and in vivo [20, 21, 28–30], were to a large extent shared among the isolates of Genome Content Group 4, but absent in Genome Content Groups 1 and 3 (Fig. 3B). Isolates of Genome Content Groups 1 and 3 contained the *pdu*-*cbi-cob-hem* gene cluster encoding for 1,2 propanediol and glycerol utilization, which were absent in most strains of the Genome Content Groups 2 and 4 (Fig. 3B). This cluster mediates cross-feeding between *L. reuteri* and microbes producing 1,2-propanediol in the gut [31] as well as the production of the antimicrobial compound reuterin [32].

Strains from different hosts that cluster in the same Gene Content Group, i.e., bird versus human isolates of Gene Content Group 1, pig versus primate isolates of Gene Content Group 2, and herbivore versus human isolates of Gene Content Group 3, showed very few differences in accessory genes (Fig. 3A). Notable exceptions were genes for citrate utilization that were present in human but not in poultry isolates of Gene Content Group 1 and in herbivore but not in human isolates of Gene Content Group 3 (Fig. 3B). Genes for molybdenum cofactor (Moco) biosynthesis as well as the histidine kinase oxygen sensor NreB were identified in swine but not in primate isolates of Gene Content Group 2. The urease gene cluster (*ure*), which is very consistently detected in rodent strains and contributes to acid resistance during gastric colonization [21], was present in herbivore but not in human isolates of Gene Content Group 3 (Fig. 3B).

### Gene gain and loss during the evolution of *L. reuteri*

**Fig. 4.**
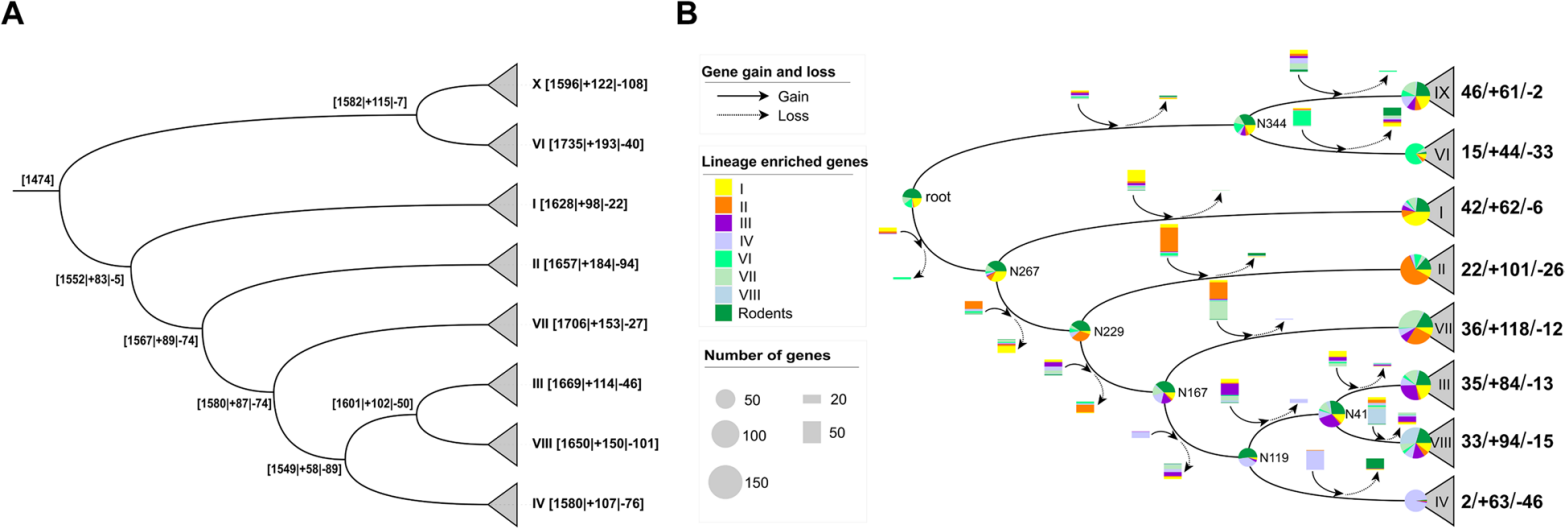
Reconstruction of the content of the ancestral genome of *L. reuteri*. **A** Gene gain and loss during the evolution of *L. reuteri*. The numbers in the square brackets represent the estimated ancestral genes, gain (“ + ”) and loss (“ − ”) events, respectively. **B** Turnover events of lineage-specific genes. Gene families with both sensitivity and specificity above 70% to certain lineage or host predicted by Scoary [33] were included. The gene gain and loss events and the ancestral status of each gene along the tree were inferred by BadiRate [34]. Circles at the end nodes represent the number of lineage-specific genes. Circles on the internal nodes refer to the numbers of estimated ancestral lineage-specific genes. Different colors of the circles represent different lineages, with the distribution of each sector proportionable to the number of genes. The oriented arrows along the tree branches stand for lineage-specific gene flow events, with solid and broken ones for gain and loss respectively. Rectangles along with the arrows represent the number of genes affected, with different color for different lineages. The three numbers at the end of the tips represent the number of genes inherited from the last common ancestor (LCA) of the species, gained during evolution, and lost compared to the LCA

To decipher the mechanisms of genome evolution that caused differences in gene content among lineages and how this relates to the clustering of Gene Content Groups (Figs. 2 and 3), gene gain and loss events were predicted for lineages with more than five isolates (Fig. 4). The last common ancestor (LCA) of *L. reuteri* was predicted to contain fewer genes than contemporary isolates, indicating a genome expansion during the evolution of *L. reuteri* (Fig. 4A). We then examined the turnover processes of the 555 lineage-specific genes. More than 90% (507/555) of these genes were acquired during the evolution of the different lineages (Fig. 4B). However, the proportion of gained genes to the total number of lineage-specific genes varied among different lineages, ranging from 57% (61/107) in lineage IX to 97% (63/65) in lineage IV. The remaining 48 lineage-specific genes were predicted to be contained by the LCA, with 28 of them shared by all rodent lineages and 20 of them retained in some but not all rodent lineages. The non-rodent lineages lost most of these genes during their evolution (Fig. 4B). Taken together, the genes shared by the rodent-associated lineages of the Genome Content Group 4 were likely inherited from a rodent-associated LCA. This assumption is also supported by the genome synteny analysis (Additional file 4: Fig. S5). The specific genes present in rodent isolates generally share similar upstream and downstream gene contexts, even among different lineages.

### Distribution of prophages among lineages of *L. reuteri*

**Fig. 5.**
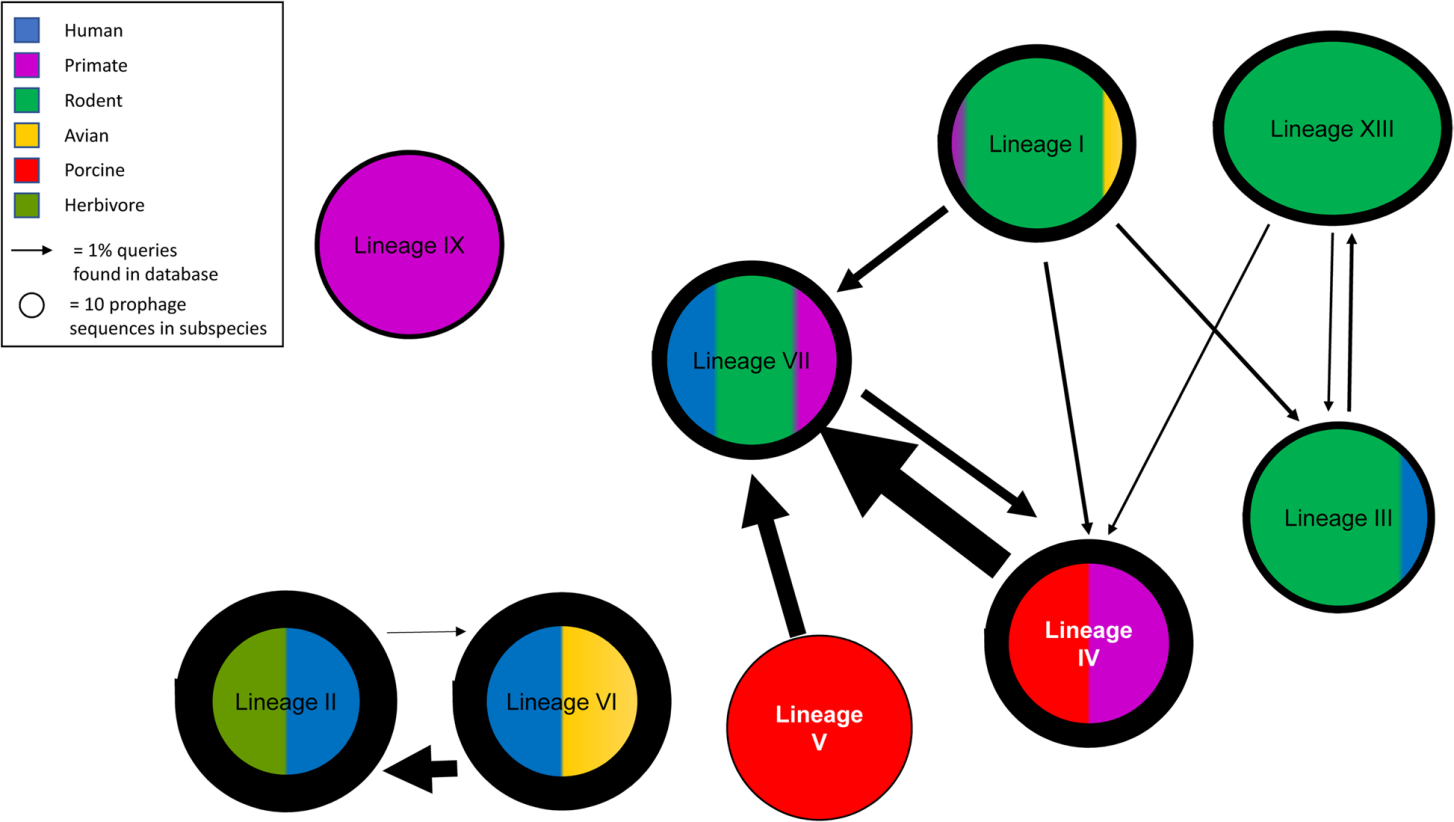
Inter-lineage exchange of lysogenic phages. Circles represent the different lineages of *L. reuteri*. The colors represent the proportion of the hosts that the lineages that have been isolated from. The width of the lines indicates the number of prophage sequences found in each of the lineages. The direction of the arrows indicates that sequences from the originating lineage that are used to generate the BLAST query database are also found in the destination lineage; the width of the arrow indicates the percent of prophage sequences from originating lineage that are found in the destination lineage. Note that the number of prophage sequences in the lineages and the arrows indicting inter-lineage exchange of prophage genes are scaled differently

Exchange of lysogenic phages, along with exchange of plasmids and transposons, represents a mechanism of horizontal gene transfer (HGT) between bacteria [35]. The analysis of phage exchange provides insight into barriers to horizontal gene exchange, which likely also represent ecological boundaries. The genome dataset for strains of *L. reuteri* was interrogated to determine whether the exchange of prophage genes is specific to lineages or hosts. The number of shared prophage genes among strains of the same lineage was more than 10 times higher than the number of prophage genes that were shared among strains of different lineages (Fig. 5). The median intra-lineage ANI is only about 1–2% higher than the median inter-lineage ANI (Fig. 1B), and lineage IX did not share any prophages with other lineages (Fig. 5), suggesting that prophages are not predominantly acquired by vertical inheritance but shared by horizontal gene transfer among closely related strains sharing the same habitat. Irrespective of their phylogenetic relatedness, strains of the rodent lineages were much more likely to exchange prophage genes among each other when compared to strains isolated from other hosts (Fig. 5). Likewise, strains of lineages II and VI, both of which contain human isolates but are not more closely related to each other than to other lineages, exchange prophage genes among each other but not with strains of other lineages. Strains of lineage VII, which were isolated from diverse rodent, humans, and non-human primate hosts, exhibited the highest frequency of exchange of prophage genes with strains from other lineages (Fig. 5).

### Rodents are the ancestral hosts of *L. reuteri*

**Fig. 6.**
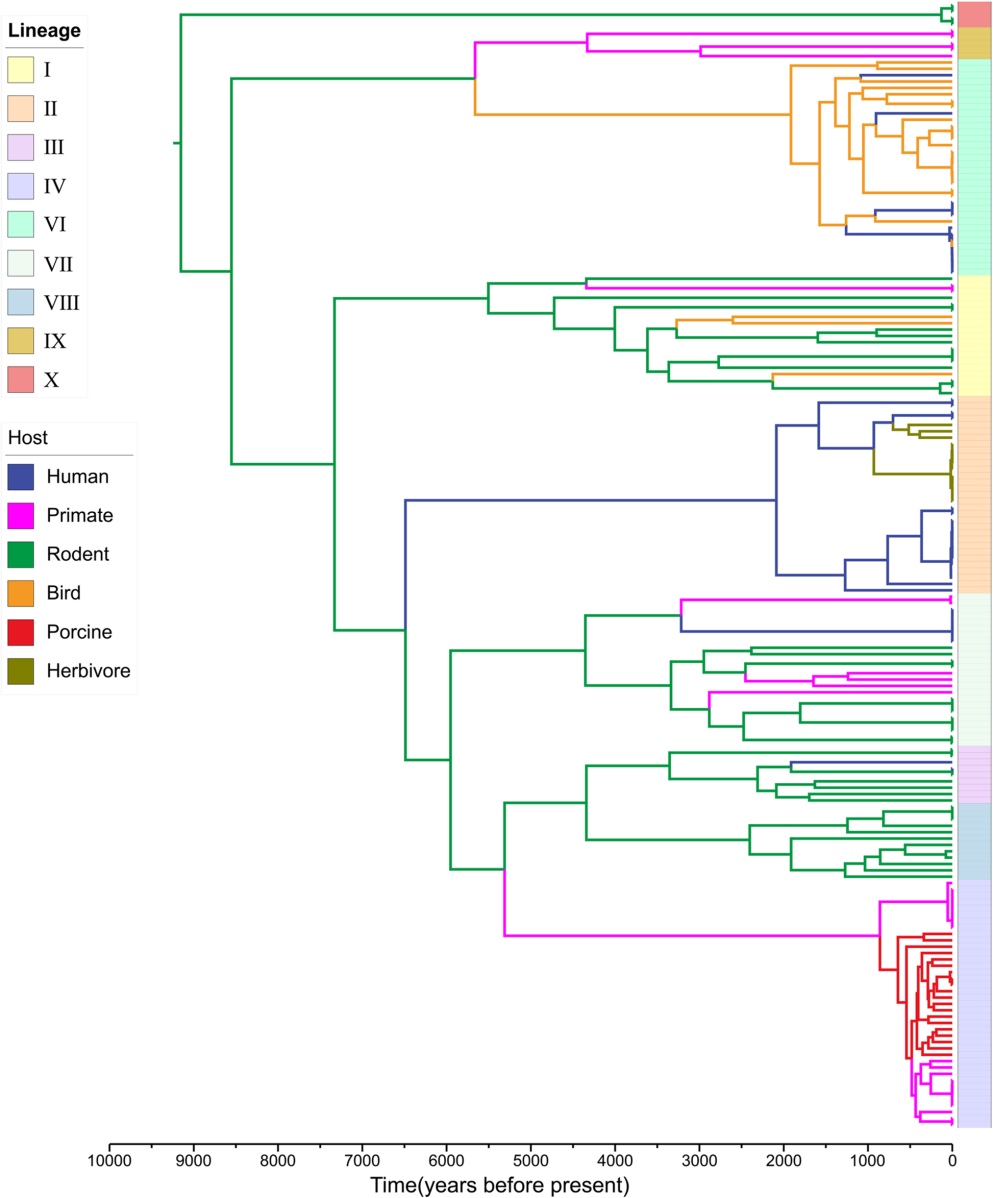
Time-scaled evolutionary history of host switches. Time-scaled phylogeny of the 182 strains of *L. reuteri*. Branches are colored according to the inferred ancestral host-species group. Vertical color strips indicate population structure (lineage I to IX)

To gain insight into the evolutionary history of *L. reuteri*, we constructed the time-scaled phylogeny and inferred the ancestral host association based on the core genes using BEAST [36] (Fig. 6). Using *Limosilactobacillus fermentum* as outgroup, a molecular clock rate of 3 × 10^−6^ substitutions per site per year was estimated for *L. reuteri* (Additional file 4: Fig. S6). Bayesian phylogenetic analysis estimated that divergence of *L. reuteri* from its LCA to multiple lineages dates back about 9100 years ago (95% highest posterior density interval [HPD]: 9000–9300). According to the host ancestral state analysis, rodents were predicted as the ancestral hosts of *L. reuteri*. Three milestone host transition events of *L. reuteri* occurred between 7000 and 5000 years ago: transitions from rodents to humans and herbivores (lineage II); and transitions from rodents to birds (lineage VI) and primates (lineage IX). Human strains in lineage VI emerged more recently than bird and primate strains; transitions from rodents to non-human primates and pigs (lineage IV) were estimated to have occurred about 5300 years ago (95% HPD: 5300–5400) (Fig. 6).

### Epithelial colonization of forestomach in germ-free mice

**Fig. 7.**
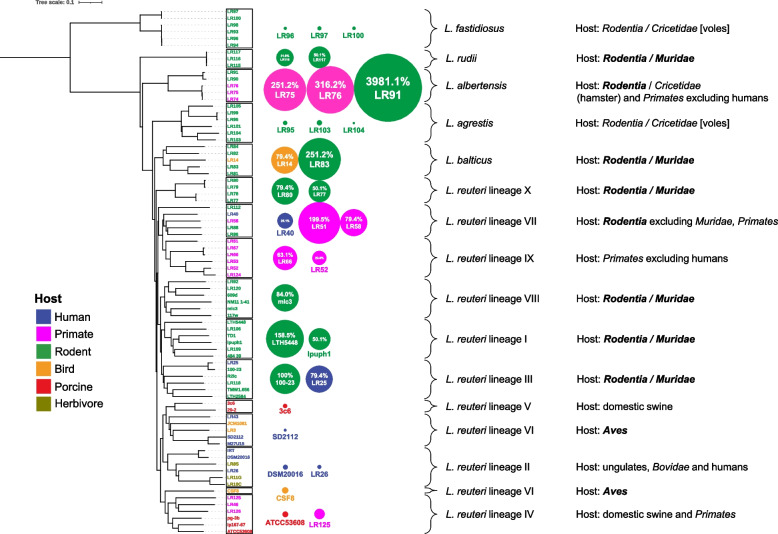
Colonization of the forestomach of germ-free mice by *L*. *reuteri* and related *Limosilactobacillus* species. The ML phylogenetic tree was constructed based the core-genome alignment of the representative strains from each of ten *L. reuteri* lineages and five *Limosilactobacillus* species. Adherent cell numbers of strains are represented by the area of the bubble besides the tree. The area of the bubble is scaled to be proportional to the log transformed cell counts that are expressed as % relative to strain *L*. *reuteri* subsp. *rodentium* 100–23. The cell counts are also shown in Additional file 4: Table S4 (*L*. *reuteri* strains) and Additional file 4: Table S5 (other five *Limosilactobacillus* species). Cell densities of more than 6.9 log10 of CFU/g, 20% relative to *L*. *reuteri* subsp. *rodentium* 100–23, were considered as effective epithelial adhesion; cell densities of less than 10%, corresponding to less than 6.6 log10 of CFU/g, were considered as ineffective epithelial adhesion. Shown to the right are the sources of isolation of strains of the respective linages. Taxonomic ranks of the respective hosts are printed in bold if host adaptation was demonstrated experimentally in this study or previously [17, 18, 39]

Biofilm formation in the forestomach is a key characteristic of the niche occupied by *L. reuteri* in rodents [37, 38], and the ability to adhere and establish biofilms is a key determinant of the competitiveness of *L. reuteri* in this host [16, 17]. In previous studies, only *L. reuteri* strains from rodent-associated lineages adhere efficiently to the forestomach epithelium in mice and form gastric biofilms [17]. Epithelial adherence thus can be used to experimentally validate the adaptation of *L. reuteri* strains to rodents, and thus a key ecological consequence of the evolutionary process. Therefore, we tested the ability of 18 strains of *L. reuteri* (Fig. 7; Additional file 4: Table S4) and 13 strains of closely related species (Fig. 7; Additional file 4: Table S5) to colonize the forestomach of ex-germ-free mice. All strains of the Genome Content Cluster 4 (Fig. 2) showed high (≥ 10^6.9^ CFU/g, corresponding to ≥ 20% relative to *L. reuteri* subsp. *rodentium* 100–23) levels of epithelial colonization in the forestomach of mice, irrespective of their source of isolation (Fig. 7; Additional file 4: Table S4). In contrast, strains from other clusters and of the lineages IV, II, V, and VI showed low (≤ 10^6.6^ CFU/g, corresponding to ≤ 10% relative to *L*. *reuteri* subsp. *rodentium* 100–23) colonization levels (Fig. 7; Additional file 4: Table S4). This analysis convincingly links Genome Cluster 4 to epithelial adherence in mice, suggesting its role as a selective force in the evolution of distinct phylogenetic lineages. Of the closely related species, strains of *L*. *balticus*, *L. albertensis*, and *L*. *rudii* showed high levels of colonization of the forestomach epithelium of mice, while *L*. *fastidiosus* and *L*. *agrestis* did not (Fig. 7; Additional file 4: Table S5). As most strains of closely related species of *L*. *reuteri* were isolated from rodents [19, 39] and colonize germ-free mice (Fig. 7), this suggests that the association of *L*. *reuteri* with rodents might have predated the emergence of this species.

## Discussion

By combining phylogenetic and gene content analysis of strains of *L. reuteri* from multiple hosts and closely related species with mouse experiments to study the consequences of evolution, our findings provide insight into the mechanisms by which a gut microbe evolves with its vertebrate hosts. Most importantly, we provide evidence for a long-term stable evolutionary relationship of a bacterial symbiont with rodents that likely even predated the emergence of the bacterial species. While *L. reuteri* diversified into several lineages that stayed associated with rodents, remained functionally similar, and maintained the ability to colonize mice, other lineages became associated with different vertebrate hosts and lost genetic colonization factors and the ability to colonize rodents. Several lineages that emerged more recently are associated with swine, non-human primates, and herbivores, and their ecological strategy is likely less host specific. Human isolates cluster with poultry strains in lineage VI and herbivores in lineage II, suggesting a more dynamic lifestyle that employs multiple hosts and the reliance of transmission between hosts (dispersal).

To understand the ecological forces shape bacterial evolution, it is valuable to consider ecological niche concepts in the context in which adaptive evolution occurs [40]. More specifically, one has to differentiate establishment niches, where a bacteria population maintains a positive growth rate even if rare and isolated from the larger species pool, from persistence niches where a bacterial population persists only temporary and is reliant on dispersal (immigrants) [40]. In short, the source of isolation does not always reflect the establishment niche. The mismatch between the source of isolation and the establishment niche has been amply documented for zoonotic pathogens, which adapted to livestock animals but temporarily persist and cause disease in humans [41]. For *L. reuteri*, it was documented that isolates from sourdough are equally competitive in mice as rodent isolates [42]. It is a strength of *L. reuteri* as a model allowing the experimental test of host adaptations to help in interpreting findings from phylogenomic analyses. In addition, we include ecological considerations in our interpretation below, such as the prevalence and temporal stability of *L. reuteri* populations in different hosts.

Multiple lines of evidence point to stable evolutionary relationships of *L. reuteri* lineages with rodents, which would provide an establishment niche. Rodent-adapted lineages of *L. reuteri* consistently clustered in Gene Content Group 4 and were therefore functionally similar. Our results indicate that the genes shared by the rodent-adapted lineages are ancestral genes inherited from last common ancestor of *L. reuteri*, while most of these genes were lost in the non-rodent lineages during the evolution. Our mouse experiments further confirmed that these rodent lineages are functionally equivalent in terms of colonization of mice because strains of these lineages efficiently colonize the mouse forestomach that is a fundamental basis for competitiveness in the rodent gut [17, 20, 39]. These rodent lineages are genetically more diverse than other lineages, suggesting longer timelines for evolution and diversification. Strains from lineage X, which cluster most closely to the basal node of *L. reuteri* and closely related species, were isolated from rodents and can also colonize forestomach epithelia in mice, suggesting that the association with rodents predates the emergence of the species *L. reuteri*. Therefore, although a recent study [10] questioned the stable associations between host-restricted strains and host species and showed that they are vulnerable to environmental change (captivity), our findings demonstrated that *L. reuteri*, a gut symbiont of vertebrate hosts, is stably associated with rodents over long evolutionary time spans and maintained, at high levels, in virtually all mouse colonies studied [43].

Rodent-specific lineages of *L*. *reuteri* also inform the boundaries of host specificity, as these include isolates from *Muridae* (lineages I, III, VIII, and X) or other families in the order *Rodentia* (lineage VII). Isolates of *L. reuteri* that clustered with the Genome Content Cluster 4 isolated from rodent families other than the *Muridae* also colonized mice, indicating the host specificity of *L.* *reuteri* may be at the family or order level for rodents. However, *L.* *fastidiosus* and *L*. *agrestis*, which originate from voles (rodent family *Cricetidae*), did not colonize mice. Because our set of strains includes multiple strains from wild animals (yellow highlighted strains in Additional file 1: Table S1), results are not confounded by “humanization” of gut microbiota of domesticated or laboratory animals [10]. The broad host specificity at the host family or order level may relate to physiological similarities among rodents, which does not exert selective pressure for niche differentiation of *L*. *reuteri* to specific rodent hosts. For *L*. *reuteri*, the boundary of host specialization, or in ecological terms, the range limit of niche space [40], lies between host orders or families. Most importantly for the hologenome concept, strict co-diversification, as it has been observed for strictly vertically inherited symbionts of insects [7], does not seem to apply to *L*. *reuteri*.

Our phylogenetic analysis revealed that the associations of lineage VI with birds occurred early in the evolution of *L*. *reuteri* and that the association remained evolutionarily stable. The inclusion of isolates from undomesticated birds did not disrupt the lineage that was previously identified using only isolates from poultry [15, 16, 18]. Most isolates were obtained from birds in the order *Galliformes*, but host species of other orders of the class *Aves* were also represented. In competition with strains of other lineages, strains of lineage VI, even if isolated from humans, showed enhanced ecological fitness in chicken while such an increased ecological fitness was not observed in humans [18]. In addition, *L*. *reuteri* is prevalent and dominant in poultry [44], while rarely detected in humans [45]. These suggest that this lineage is indeed adapted to birds but not to humans. Overall, our findings indicate that lineage VI of *L*. *reuteri* evolved for a sufficient time separately from rodent-adapted strains to associate with birds as the establishment niche [40], and the boundary of host specificity of *L. reuteri* appears to lie at the order or class level.

The evolutionary strategies and lifestyles of other *L*. *reuteri* lineages are less clear. Lineage IX is composed of only five isolates from non-human primates and therefore under-represented in our set of isolates and genomes. Lineage VII contains isolates from humans, non-human primates, and rodents. Gene content analysis assigns these two lineages to Genome Content Group 4, showing that these two lineages share functional genes with other rodent-dominated lineages and possess genetic factors for rodent colonization. In fact, strains from lineages VII and IX colonized the forestomach of ex-germ-free mice, suggesting that these strains are autochthonous to rodents. Most primate isolates cluster with porcine isolates in lineage IV, which was previously composed only of swine isolates [15, 16]. This lineage emerged very recently, and swine isolates from lineage IV showed no evidence for adaptation or higher ecological performance in colonization experiments in pigs [46]. Strains are either from domesticated pigs or zoo primates, which may confound the analysis as described by Nishida and Ochman [10], and point to a recent acquisition due to domestication or the captive environment. In fact, we were unable to isolate *L*. *reuteri* from wild boars and pig species from zoos, and the species was rarely detected in wild pigs [46, 47]. Our comparative analysis also did not detect major differences in gene content between non-human primate and swine isolates of lineage IV. Overall, the dataset presented in this study indicates that *L*. *reuteri* may be a recent acquisition to the gut microbiota of non-human primates and swine driven by domestication, and this recent association was not sufficient for host-specific adaptation.

*L. reuteri* is often considered autochthonous to humans [48, 49], and host-restricted phylogenetic clusters of human strains were identified in previous studies [15, 16]. However, our work here shows that human *L*.* reuteri* strain cluster tightly with herbivore and bird isolates of lineage II and VI, respectively, and human strains in these lineages do not possess human-specific genes compared to strains from birds and domesticated herbivores that cluster in the same lineages. This suggests that the human strains in lineages II and VI do not differ in their lifestyle or ecological strategy from poultry and herbivore strains, questioning a stable association with humans. This conclusion is supported by a range of consistent observations. First, *L*. *reuteri* is rarely isolated from contemporary humans [50, 51], and a large-scale analysis of human fecal microbiomes (9445 metagenomes) showed low prevalence and abundance of *L*. *reuteri*, suggesting that the species is not a long-term resident [45]. Second, human strains of lineages II and VI do not show elevated ecological performance in competition experiments in humans, while lineage II strains, independent of origin, are competitive in chicken [18]. Human strains in lineage VII cluster, which originate from human living in rural Papua New Guinea, cluster with rodent strains in Gene Content Group 4 and show colonization of mouse forestomach epithelium, strongly suggesting they are autochthonous to rodents. Overall, these findings question the autochthony of *L*. *reuteri* in humans and suggest chicken, herbivores, and rodents as the establishment niches of *L*. *reuteri* and that strains isolated from humans were acquired from domesticated animals or rodents. It seems that transmission repeatedly occurred in different geographic locations because human strains analyzed in this study were repeatedly isolated from various locations at different times.

Experimental assessment for bacterial mutation rates (substitutions per site and year) ranges from less than 10^−8^ to more than 10^−6^ even for closely related bacterial species or strains [52, 53]. A molecular clock rate of 3 × 10^−6^ was estimated in this study, which corresponds to the clock rates that were used to estimate the time-scaled evolution of *Campylobacter* *jejuni* and *Staphylococcus* *aureus* [54, 55]. However, confident dating of bacterial evolution is impossible in the absence of an archeological record. Despite this uncertainty, our results suggest that the timelines of transmission of *L*.* reuteri* to hosts other than rodents, birds and possibly herbivores, match the time of early human settlements and the domestication of animals about 9000 years ago [56–58]. The storage of food and feed in human settlements attracts rodents and thus increases the exposure of humans to rodents, domestic animals, and their commensal microbes.

Our work complements recent efforts to elucidate the evolutionary relationships of vertebrate gut symbionts with their hosts [5, 9, 10], and how they are influenced by ecological processes such as dispersal (transmission) [59]. This work has shown that lifestyles of vertebrate gut symbionts can range from species stably associated with hosts (with signs of co-diversification, showing genomic adaptations of host dependency, i.e., reduced genome size and oxygen and temperature sensitivity), to species more loosely associated with hosts and mainly relying on dispersal strategies [5]. Our present work shows that the diversity of lifestyles, in a host-dependent manner, can be represented within a single bacterial species, ranging from strictly host confined in rodents and birds, to allochthonous possibly reliant on zoonotic transmission in humans. In addition, scientists have voiced caution that ecological processes, such as geographic separation and environmental factors (i.e., diet), can generate host-restricted bacterial clades and mimic patterns of co-diversification that do not persist when environmental pressures change [10]. We argue that studies on model organisms such as *L. reuteri* can be used to confirm findings from phylogenomic analyses to shed light into the complex mechanisms by which gut symbionts evolve and how this process is impacted and interacted by environmental pressures.

Apart from providing basic information on the evolution of a gut symbiont, our work has relevant practical implications that relate to health. *L. reuteri* was frequently isolated from humans in the middle of the twentieth century [49]. The ongoing transmission of *L. reuteri* to humans that was discovered in this study is relevant, as the species has been shown to be health-promoting, immunomodulating, and anti-inflammatory [60–62]. Of note, studies that documented the health effects of *L. reuteri* often used strains that are allochthonous to the respective host that was studied. For example, an autism prevention study with mice as the experimental model used *L. reuteri* ATCC-PTA-6475 [61], which is of human origin. Therefore, zoonotic transmission might have health consequences. Close contact between humans and domestic animals is known to result in exchange of zoonotic or anthropozoonitic pathogens [54, 55]. Zoonotic transmission of gut symbionts, however, may be beneficial for human health [63]. Living on farms, and thus frequent contact with poultry, herbivores, swine, and likely rodents, has been consistently linked in the epidemiological literature with health benefits (e.g., allergies, autoimmune diseases) [64, 65]. The transmission of *L. reuteri* from animals would explain why the species seems to become reduced through industrialization and urbanization [28]. The reduced transmission of zoonotic symbionts is thus a possible or even likely contributor to the depletion of the microbiome in humans that live in urbanized and industrialized societies [66]. Therefore, a further enhanced insight into the health effects of the zoonotic symbiont transmission will be the foundation for developing intervention strategies, for example with probiotics or live dietary microbes, for manipulating the gut microbiome to improve health.

## Conclusions

This study provides key insights into the evolution of a gut symbiont, demonstrating the complexity of evolutionary processes, ecological patterns, and lifestyles even just within a single bacterial species. Previous studies formed speculations on co-speciation of hosts and their symbionts predominantly on the basis of a congruence phylogeny of host species and their symbionts [2, 9]. We expand the toolset for analysis of host-symbiont co-evolution by additionally considering the gene content, mechanisms of gene exchange, gene loss and gain, and an experimental validation of host adaptation. Lifestyles can vary from highly host-specific to dynamic and transient. Although some aspects of *L. reuteri*, such as the stable association with rodents and birds over evolutionary times, might fulfill characteristics of the hologenome hypothesis, the diversification into different linages was associated with ongoing horizontal gene transfer and host switches. Some vertebrate hosts clearly provide an establishment niche as documented by stable associations over evolutionary timelines, but other hosts merely serve as persistence hosts or as temporary habitats. An understanding of these relationships contributes to our ability to use these microbes to improve health.

## Methods

### Isolation of strains

The strain collection of *L. reuteri* used in previous studies was composed of strains primarily isolated from humans, domestic chickens and turkeys, lab mice, and domestic pigs [15, 16, 18, 19]. In the present study, we isolated *L. reuteri* strains from a wider phylogenetic assortment of zoo and wild animal hosts (e.g., six species of birds, nine species of non-human primates, and eight species of rodents) and humans from both industrialized and non-industrialized societies. In brief, a total of 94 *L. reuteri* strains isolated from fresh feces or gut samples of multiple vertebrate hosts (*n* = 19 from birds, *n* = 32 from non-human primates, *n* = 23 from rodents, and *n* = 20 from humans) and 25 strains belonging to *Limosilactobacillus balticus* (*n* = 5), *Limosilactobacillus agrestis* (*n* = 6), *Limosilactobacillus albertensis* (*n* = 5), *Limosilactobacillus rudii* (*n* = 3), and *Limosilactobacillus fastidiosus* (*n* = 6) were selected for the whole-genome sequencing in the present study (Additional file 1: Table S1). Strains were cultured anaerobically either on MRS (De Man, Rogosa, Sharpe) or modified MRS (mMRS; MRS supplied with 10 g/L maltose and 5 g/L fructose) medium at 37 °C unless otherwise noted. Detailed information for the source of isolation and genome accession numbers of these strains is shown in Additional file 1: Table S1.

We also attempted to isolate strains of *L. reuteri* from samples of zoo and wild pigs. We collected fresh fecal samples of peccaries, Vietnamese pot-bellied, red river hog, and wild boar, from Greater Vancouver Zoo (Vancouver, BC, Canada), Calgary Zoo (Calgary, AB, Canada), Edmonton Valley Zoo (Edmonton, AB, Canada), Assiniboine Park Zoo (Winnipeg, MB, Canada), and Omaha’s Henry Doorly Zoo and Aquarium (Omaha, NE, USA). However, these attempts were not successful and none *L. reuteri* strain was isolated from pigs.

### Genome sequencing, assembling, and annotation

Genomic DNA of each bacterial strains was extracted from the overnight cultures with the Wizard Genomic DNA Purification Kit (Promega Corporation, Madison, WI, USA), following the manufacturer’s protocol for Gram-positive bacteria. Sequencing libraries were constructed using the NEBNext Ultra II DNA Library Prep Kit (New England Biolabs Ltd., Whitby, ON, Canada), and sequencing on the Illumina HiSeqX platform was provided by service of the Génome Québec Innovation Centre (Montréal, QC, Canada). The quality of sequencing outputs was assessed using the FastQC program (https://github.com/s-andrews/FastQC). Trimmomatic v0.36 [67] and Quake [68] were used to filter residual adapters and trim bases with low quality. Most of the downstream analyses were then performed using the prokaryotic genomic analysis platform (https://github.com/BMBGenomics/pgcgap). Briefly, for each genome, post-QC reads were assembled using SPAdes [69] with the k-mer lengths of 21, 33, 55, 77, 91, and 105. Contigs classified as phi-X174 (NCBI accession: NC_001422.1) were eliminated, and scaffolds with length less than 200 bp were discarded. The assembled draft genomes were annotated with Prokka [70] that uses Prodigal [71] for gene prediction. Publicly accessible *L. reuteri* genomes were reannotated with the same tools. Protein sequences were functionally annotated using the eggNOG 5.0 database [72] and eggNOG-mapper v2 (https://github.com/eggnogdb/eggnog-mapper/wiki).

### Pan-genome analysis and phylogeny inference

In addition to the 94 genomes sequenced in the present study, 88 *L. reuteri* genomes available in public databases were also retrieved and included for analyses, and their accession numbers can be found in Additional file 1: Table S1. The pan-genome analysis was carried out using Roary v3.6.1 [26] based on the GFF3 files generated from Prokka [70], with default parameters except for setting the minimum percentage identity for Blastp as 90%. This parameter was set to 80% when genomes of *L. agrestis* were used as outgroup. For the pan-genome analysis of the genus *Limosilactobacillus*, in addition to *L. reuteri* genomes, 25 isolates belonging to 5 closely related species [19] isolated from zoo or wild rodents were further included (Additional file 1: Table S1). Orthologous clusters were computed by OrthoMCL [73] using the FastOrtho tool (https://github.com/PATRIC3/FastOrtho). Briefly, protein sequences of all the selected genomes were searched through an all-against-all method by Blastp with an *e*-value below 10^−10^, and then clustered by the Markov Cluster Algorithm (MCL) with an inflation value of 2. Core genes, which are present in more than 95% of the total genome dataset, and accessory genes were extracted from the cluster results.

To construct the phylogenetic tree for *L. reuteri*, coding sequences of single-copy core genes were extracted according to the cluster results generated by Roary [26] and aligned using MUSCLE [74]. Recombination events were predicted using PhiPack [75] with 100 permutations based on each alignment with a *p*-value of 0.05 as cut-off. Sequence alignments without recombination events were trimmed using TrimAl [76] and concatenated into a new alignment. Maximum likelihood (ML) phylogenetic analysis was then conducted based on the recombination-free core gene alignment using RAxML-NG [77], with the best nucleotide substitution model (GTR + I + G4) estimated from ModelTest-NG [78] and 1000 bootstrap replicates. Population structure was defined based on the recombination-free core gene alignment using the BAPS (Bayesian Analysis of Population Structure) built-in module hierBAPS [25], which delineates the population structure through nested clustering. The inferred phylogenetic tree was visualized using iTOL [79]. For the phylogenetic analysis of the genus *Limosilactobacillus*, protein sequences of single-copy core genes were aligned by MUSCLE [74], filtered through TrimAl [76], and concatenated as descripted above; the ML tree were constructed using RAxML-NG [77] with 500 replicates of non-parametric bootstrapping.

### Genome similarity and dissimilarity analysis

Pairwise average nucleotide identity (ANI) was calculated between whole-genome sequences using fastANI [80]. Core-genome SNP distance of each genome pair was obtained via SNP-dist (https://github.com/tseemann/snp-dists) based on the alignment of core genes. The Jaccard distance (JD) between each genome pair is defined as 1 − (A ∩ B)/((A ∪ B) − (A ∩ B)), where (A ∩ B) is the number of shared gene, and (A ∪ B) is the number of total genes of each genome pair. The JD matrix was calculated based on the accessory genes present in less than 95% of the total genome dataset but in more than one genome. Data of ANI, core gene distance and JD were plotted by the Python data visualization library seaborn (https://seaborn.pydata.org). Nonmetric multidimensional scaling (NMDS) of gene content based on the JD matrix was conducted using the metaMDS function from the vegan package (https://github.com/vegandevs/vegan) in R (https://www.r-project.org/). The gene content tree was inferred based on the JD matrix through the MEGA-X build-in UPGMA method [27] and visualized using iTOL [79].

Pan-genome-wide association studies (Pan-GWAS) were applied to predict lineage- and Gene-Content-Group-specific genes using Scoary [33]. If an *L. reuteri* isolate belongs to a certain lineage or gene content group, it was coded as 1; otherwise, it was coded as 0 as the binary-category trait information, which were then associated with gene presence and absence pattern with 1000 permutation replicates in Scoary [33]. Genes were considered lineage- and Gene-Content-Group-specific only if all three types of *p*-values (naive, Benjamini-Hochberg-corrected, and empirical) were lower than 0.05, and both specificity and sensitivity were above 70%.

### Ancestral genome reconstruction and inference of gene gain and loss

The evolution of gene content via gene gain and loss was estimated using BadiRate v1.35 [34]. The gene content table and the presence/absence table of the lineage-specific gene groups were used for two independent analyses. The gene gain and loss events were inferred only for lineages with more than five isolates. The BDI-FR-CSP model with the Turnover rates-Branch model-Estimating procedure, which is stringent on estimating turnover rates, was used to estimate the turnover rates of all genes and host- or lineage-specific genes. From the gene profile at each ancestral node, gene gain and loss events for each branch were extracted, and the results were visualized using ggplot2 (https://ggplot2.tidyverse.org) and iTOL [79].

### Determination of inter-lineage exchange of prophage genes

Prophage sequences were extracted with VirSorter2 v2.1 [81], and the quality of the sequences was determined with CheckV v0.7.0 [82]. Sequences shorter than 5 kb or predicted to have bacterial genes but not viral genes in the CheckV [82] contamination output file were discarded. The remaining sequences were grouped by lineages and used as both queries and databases in a Blastn v2.9.0 [83] search, where the prophage sequences from each lineage were searched against the sequences from every other lineage. Each query that had at least one positive hit with a fragment greater than 5 kb was counted. The number of queries found in the subject database was divided by the number of queries submitted to express the number of shared prophage genes as a percentage of the total number of prophages genes.

### Construction of the evolutionary history of *L. reuteri*

Bayesian evolutionary analysis was performed using BEAST v1.10.4 [36]. In order to estimate nucleotide substitution rate within the species *L. reuteri*, we chose *L. fermentum* as the outgroup because the time of divergence between *L. reuteri* and *L. fermentum* has been reported [84]. We used the tMRCA (time to the most recent common ancestor) prior of 2.91 × 10^7^ years. The alignment of single-copy core gene sequences of representative genomes was used for analysis, based on the GTR + I + G4 model of nucleotide substitution and the strict clock model. A Markov chain Monte Carlo (MCMC) ran for 10^8^ generations with sampling every 10,000 steps, and the first 10% of states were removed. The result of effective sample sizes was checked using Tracer v1.7.1 [85] to ensure convergence. The mean of estimated nucleotide substitution rate was 3 × 10^−6^ substitutions per site per year (Additional file 4: Fig. S6). The dated phylogeny and host ancestral states were estimated using a continuous-time Markov chain (CTMC) discrete model that was applied to core gene alignment and six major host groups within *L. reuteri*. Briefly, for sequence partition, we selected the HKY model of nucleotide substitution, and for host partition we selected asymmetric substitution model of discrete trait. All strains were treated as contemporaneous and an estimated clock rate of 3 × 10^−6^ substitutions per site per year was used. The analyses ran in Markov chains of 10^8^ steps with sampling every 100 steps and the first 10% of states were discarded. The phylogenetic tree was visualized using Figtree v1.4.3 (https://github.com/rambaut/figtree).

### Bacterial adherence to forestomach epithelium in germ-free mice

Mouse experiments to test for adherence of *L. reuteri* and closely related species to forestomach epithelium in vivo were conducted with approval of the Animal Care and Use Committee of the University of Alberta (protocol no. AUP00002099), using Germ-Free Swiss Webster mice (4–20 weeks of age, male and female) and following the animal husbandry procedures as described previously [39]. The adherence to the forestomach epithelium was quantified for eighteen *L. reuteri* strains (Additional file 4: Table S4) and 13 strains of *L. balticus*, *L. agrestis*, *L. albertensis*, *L. rudii*, and *L. fastidiosus* (Additional file 4: Table S5). Briefly, each of the strains was incubated anaerobically in MRS broth at 37 °C for 16 h. Cells were harvested through centrifugation and resuspended in sterile phosphate-buffered saline (PBS) immediately prior to the experiment. Mice were assigned to cages (*n* = 5 cages per treatment), and each mouse was inoculated by gavage of a single dose of 100 μL cell suspension corresponding to about 10^8^ bacterial cells. After 72 h of colonization, mice were sacrificed using CO_2_ asphyxiation, and the forestomach was collected. The forestomach tissue was separated from the content and washed with PBS to remove the digesta. The tissue was then transferred to a pre-weighted 2-ml screw-cap tube containing 1-ml PBS and 2-mm zirconium beads. The tissue was disrupted to dislodge adherent bacterial cells by vortexing with zirconium beads for 1 min. Bacterial cell numbers were determined by surface plating on MRS agar. Results for these strains were expressed relative to adherent cell numbers of *L. reuteri* subsp. *rodentium* 100–23, which served as a positive control [16, 17]. Cell densities of more than 6.9 log10 of CFU/g, 20% relative to *L. reuteri* subsp. *rodentium* 100–23, were considered as effective epithelial adhesion; cell densities of less than 10%, corresponding to less than 6.6 log10 of CFU/g, were considered as ineffective epithelial adhesion.

## Supplementary Information

**Additional file 1: Table S1.** Genome list and metadata.

**Additional file 2: Table S2. **Average nucleotide identity values between *L. reuteri* lineages and closely related species in the genus *Limosilactobacillus*.

**Additional file 3: Table S3.** Lineage-specific accessory genes.

**Additional file 4: Figure S1.** Jaccard distance of genome pairs belonging to the same or different lineages based on accessory genes. **Figure S2.** Genome sizes of *L. reuteri* lineages that contain five or more isolates. **Figure S3.** Sizes of pan-genome (A) and core-genome (B) of different *L. reuteri* lineages. **Figure S4.** Group defined based on gene content dissimilarity (Jaccard Distance). **Figure S5.** Microsynteny comparison of rodent specific genes from different lineages. **Figure S6.** Frequency distribution of clock rates and summary of statistical value. **Table S4.** Cell numbers of *L. reuteri* strains adherent to the forestomach epithelium of germ-free mice. **Table S5.** Cell numbers of five *Limosilactobacillus* species adherent to the forestomach epithelium of germ-free mice.

## Data Availability

Genome sequences of *Limosilactobacillus reuteri* and five *Limosilactobacillus* species generated in the present study were deposited into NCBI GenBank with accession numbers PRJNA771229 [86] and PRJNA649652 [87]. The accession numbers of previously published genome sequences analyzed in this study can be found in Additional file 1: Table S1.
