# Supplementary material for: A phylogenomic analysis of Limosilactobacillus reuteri reveals ancient and stable evolutionary relationships with rodents and birds and zoonotic transmission to humans
Source: BMC Biol. 2023 Mar 13;21:53. doi: 10.1186/s12915-023-01541-1 (PMC10010030; doi:10.1186/s12915-023-01541-1)
Supplement: Supplementary file 4 — Additional file 4: Figure S1. Jaccard distance of genome pairs belonging to the same or different lineages based on accessory genes. Figure S2. Genome sizes of L. reuteri lineages that contain five or more isolates. Figure S3. Sizes of pan-genome (A) and core-genome (B) of different L. reuteri lineages. Figure S4. Group defined based on gene content dissimilarity (Jaccard Distance). Figure S5. Microsynteny comparison of rodent specific genes from different lineages. Figure S6. Frequency distribution of clock rates and summary of statistical value. Table S4. Cell numbers of L. reuteri strains adherent to the forestomach epithelium of germ-free mice. Table S5. Cell numbers of five Limosilactobacillus species adherent to the forestomach epithelium of germ-free mice. [file 12915_2023_1541_MOESM4_ESM.docx]

**Supplementary information**

A phylogenomic analysis of *Limosilactobacillus reuteri* reveals ancient and stable evolutionary relationships with rodents and birds and zoonotic transmission to humans

Fuyong Li^1,2^,

Xudong Li^3,4^,

Christopher C. Cheng^1,5^,

Dalimil Bujdoš^6^,

Stephanie Tollenaar^1^,

David J. Simpson^1^,

Guergana Tasseva^1^,

Maria Elisa Perez-Muñoz^1^,

Steven Frese^7^,

Michael G. Gänzle^1*^,

Jens Walter^1,5,6*†^,

Jinshui Zheng^3,4*†^,

^1^Department of Agricultural, Food and Nutritional Science, University of Alberta, Edmonton, Alberta, T6G 2E1, Canada

^2^Department of Infectious Diseases and Public Health, Jockey Club College of Veterinary Medicine and Life Sciences, City University of Hong Kong, Kowloon, Hong Kong SAR, China

^3^State Key Laboratory of Agricultural Microbiology, Huazhong Agricultural University, Wuhan, 430070, China

^4^Hubei Key Laboratory of Agricultural Bioinformatics, Huazhong Agricultural University, Wuhan, 430070, China

^5^Department of Biological Sciences, University of Alberta, Edmonton, Alberta, T6G 2E1, Canada

^6^APC Microbiome Ireland, School of Microbiology, and Department of Medicine, University College Cork, Cork, T12 YT20, Ireland

^7^Department of Nutrition, University of Nevada, Reno, NV, 89557, USA

^†^Jens Walter and Jinshui Zheng contributed equally to this manuscript and share senior authorship.

^*^Corresponding authors:

Jinshui Zheng, [jszheng@mail.hzau.edu.cn](mailto:jszheng@mail.hzau.edu.cn);

Jens Walter, [jenswalter@ucc.ie](mailto:jenswalter@ucc.ie);

Michael G. Gänzle, [mgaenzle@ualberta.ca](mailto:mgaenzle@ualberta.ca)

Author email: Fuyong Li, [fuyongli@cityu.edu.hk](mailto:fuyongli@cityu.edu.hk),
Xudong Li, [lixudongli008@qq.com](mailto:lixudongli008@qq.com),
Christopher C. Cheng, [cccheng2@ualberta.ca](mailto:cccheng2@ualberta.ca),
Dalimil Bujdoš, [dbujdos@umail.ucc.ie](mailto:dbujdos@umail.ucc.ie),
Stephanie Tollenaar, [tollenaa@ualberta.ca](mailto:tollenaa@ualberta.ca),
David J. Simpson, [djsimpso@ualberta.ca](mailto:djsimpso@ualberta.ca),
Guergana Tasseva, [tasseva@ualberta.ca](mailto:tasseva@ualberta.ca),
Maria Elisa Perez-Muñoz, [perezmun@ualberta.ca](mailto:perezmun@ualberta.ca),
Steven Frese, [steve.frese@gmail.com](mailto:steve.frese@gmail.com)

## Supplementary Figures

##
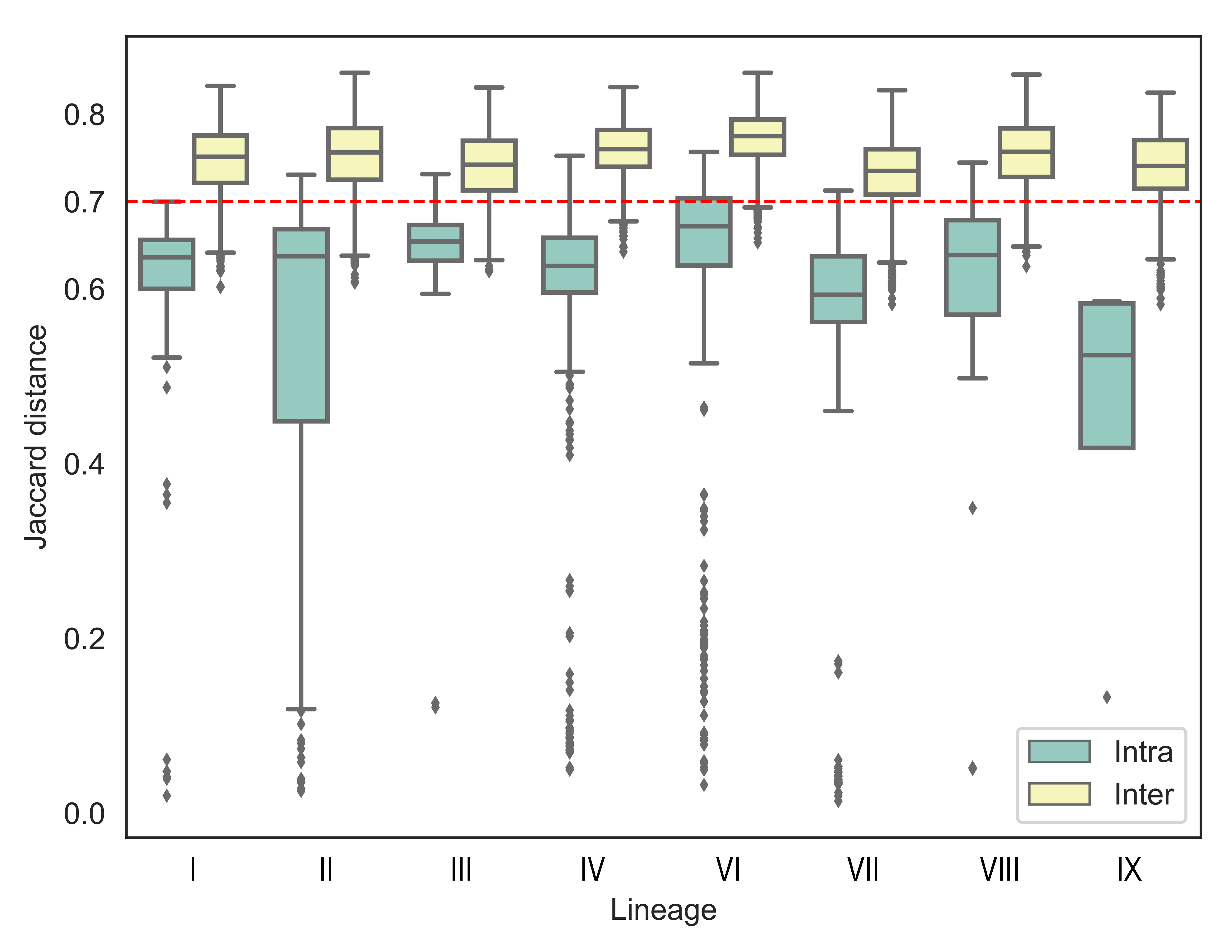


## Figure S1. Jaccard distance of genome pairs belonging to the same or different lineages based on accessory genes. Jaccard distances between different genome pairs were calculated, and only the accessory genes present in less than 95% of the total genome dataset but in no less than two genomes were included to the calculation.

**
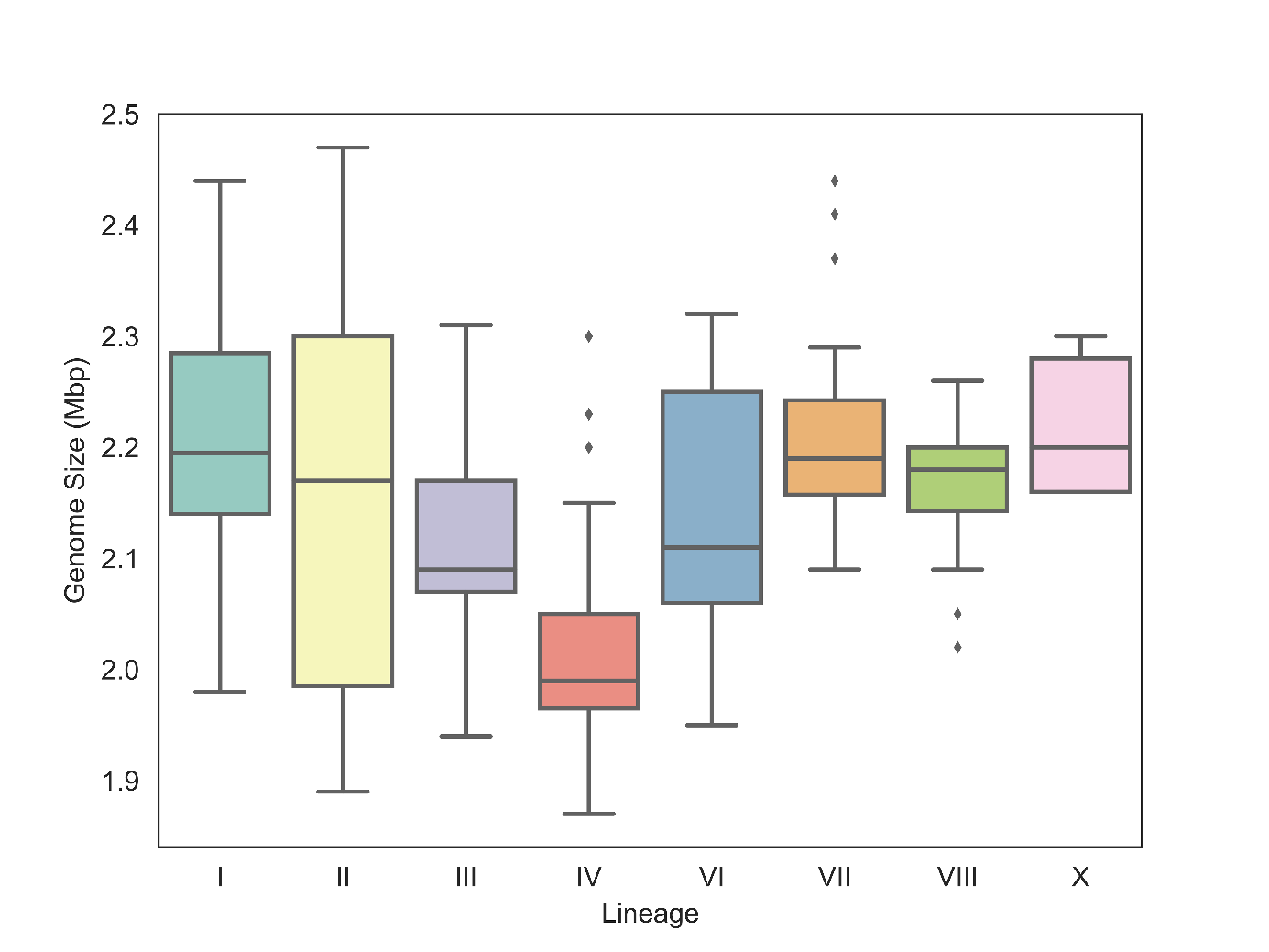
**

**Figure S2. Genome sizes of *L. reuteri* lineages that contain five or more isolates.**

**Figure S3. Sizes of pan-genome (A) and core-genome (B) of different *L. reuteri* lineages.** Pan-genome of lineage with more than 5 members was respectively analyzed by Roary [1] with an identity cut-off 90%. Randomized genome sampling was conducted 100 times in each lineage to get the average number of genes for each sample comparison number and standard deviations.

The pan- and core-genomes were computed to study the contribution of horizontal gene transfer for evolution and host adaptation of *L. reuteri* lineages. The pan-genomes of all lineages remain open, indicating continuous acquisition of genes. The number of core genes varies among different lineages. The lineage III has the smallest core genome and the largest pan-genome, while the lineage VII has the largest core genome and the smallest pan-genome.

**Figure S4. Group defined based on gene content dissimilarity (Jaccard Distance).** (A) Dissimilarity between and within four groups shown as direct Jaccard Distance plot. (B) Dissimilarity between and within four groups displayed by analysis of similarities (ANOSIM) plot. Horizontal bar in the box indicates median; bottom of the box indicates 25^th^ percentile; top of the box indicates 75^th^ percentile; whiskers extend to the most extreme data point, which is no more than the range (i.e., 1.5) times the interquartile range from the box; width of the bar is directly proportional to sample size.

**
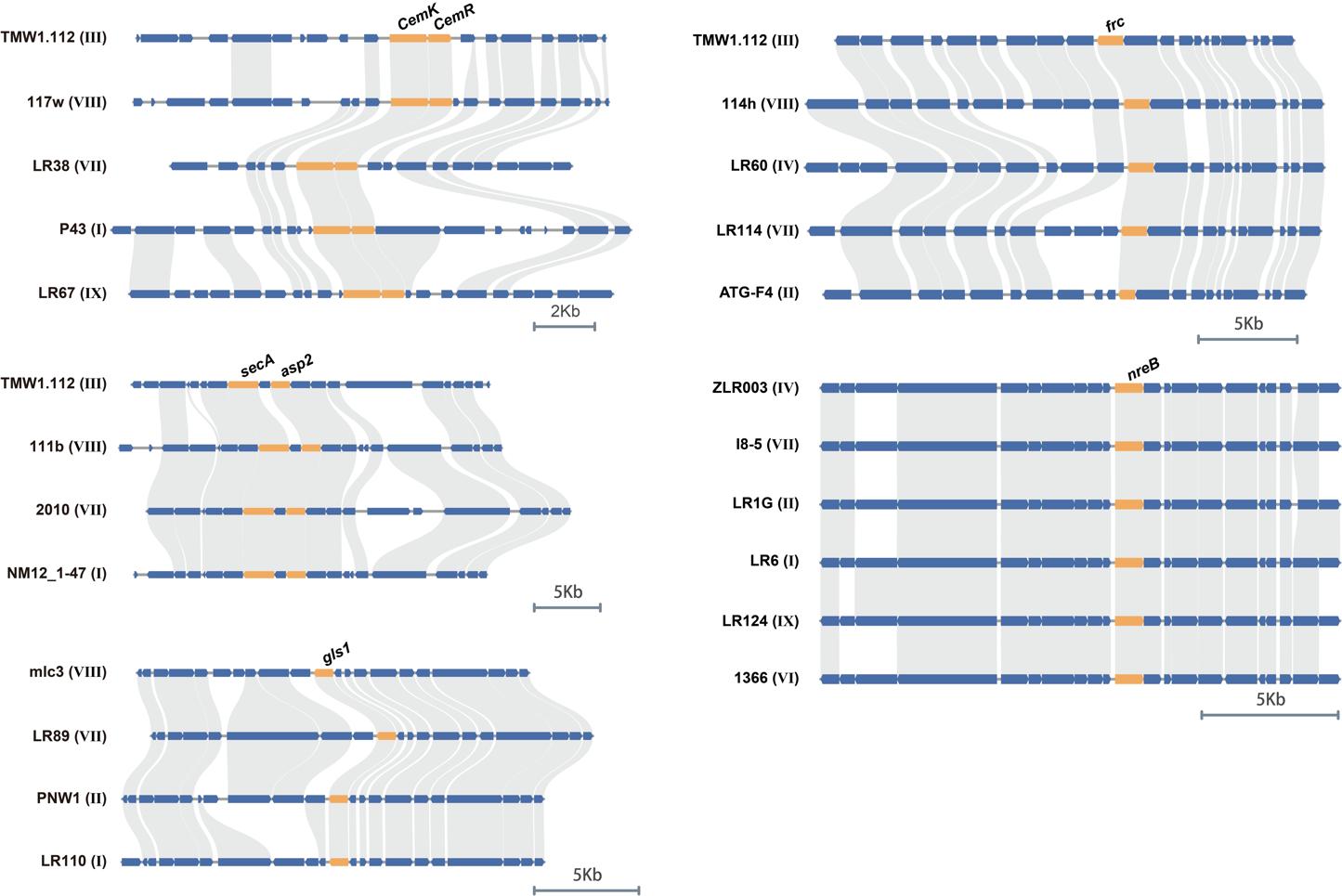
**

**Figure S5. Microsynteny comparison of rodent specific genes from different lineages**. The information of each gene is as same as described in **Fig. 3**.

**
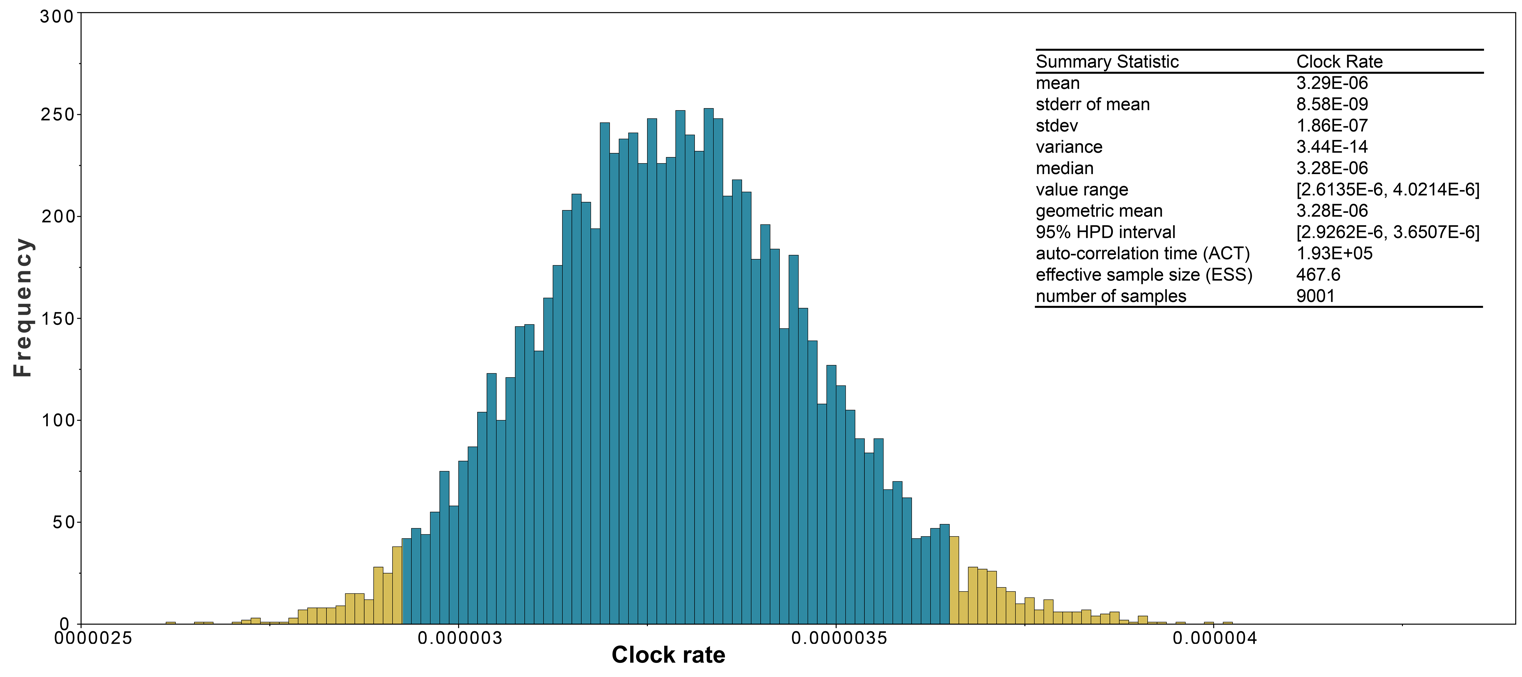
**

**Figure S6. Frequency distribution of clock rates and summary of statistical value.** The graph was generated by Tracer v1.7.1 [2] using the BEAST [3] log file. The clock rate are approximately normal distribution and various statistics is shown in the inlet. The effective sample size (ESS) is greater than 200, which means that the trace represents the posterior distribution well.

## Supplementary Tables

Table S4. Cell numbers of *L. reuteri* strains adherent to the forestomach epithelium of germ-free mice

| Strain ID/Name | Host origin | Lineage | Cell count  log_10_ (CFU/g) | % relative  to 100-23 |
| --- | --- | --- | --- | --- |
| 100-23 | Rat | III | 7.6 ± 0.4 | 100.0 |
| lpuph1 | Mouse | I | 7.3 ± 0.5 | 50.1 |
| LTH5448 | Sourdough | I | 7.8 ± 0.3 | 158.5 |
| DSM 20016 | Human | II | 5.8 ± 0.2 | 1.6 |
| LR26 (Cor137_1_1) | Human | II | 5.6 ± 0.2 | 1.0 |
| LR25 (Cor124_1_1) | Human | III | 7.5 ± 0.3 | 79.4 |
| ATCC 53608 | Pig | IV | 6.0 ± 0.5 | 2.5 |
| LR125 (LTMS_2_1) | Lion-tailed macaque | IV | 6.6 ± 0.7 | 10.0 |
| 3c6 | Pig | V | 5.7 ± 0.6 | 1.3 |
| CSF8 | Red junglefowl | VI | 6.1 ± 0.5 | 3.2 |
| SD2112 | Human | VI | 5.2 ± 0.9 | 0.4 |
| LR40 (PB-W1) | Human | VII | 7.0 ± 0.6 | 25.1 |
| LR58 (LTMS_1_1) | Lion-tailed macaque | VII | 7.5 ± 0.1 | 79.4 |
| LR51 (BHL_1_1) | Black howler | VII | 7.9 ± 0.3 | 199.5 |
| LR66 (PMP_1_1) | Patas monkey | IX | 7.4 ± 0.5 | 63.1 |
| LR52 (BHL_2_1) | Black howler | IX | 6.9 ± 0.8 | 20.0 |
| LR77 (WF-AA1-A) | Striped field mouse | X | 7.3 ± 0.4 | 50.1 |
| LR80 (CC-AA2-2) | Striped field mouse | X | 7.5 ± 0.1 | 79.4 |

Cell numbers of *L. reuteri* strains colonized on the forestomach epithelium of germ-free mice were quantified by viable plate counts on MRS agar. Germ-free mice were inoculated by gavage with a single dose of different strains 3 days before sacrifice. The colonization of the forestomach epithelium is expressed as % relative to *L. reuteri* subsp. *rodentium* 100-23, for which the biofilm formation in mice is well-characterized. Cell densities of more than 6.9 log10 of CFU/g, 20% relative to *L. reuteri* subsp. *rodentium* 100-23 were considered as effective epithelial adhesion; cell densities of less than 10%, corresponding to less than 6.6 log10 of CFU/g, were considered as ineffective epithelial adhesion.

## Table S5. Cell numbers of five *Limosilactobacillus* species adherent to the forestomach epithelium of germ-free mice

| Strain ID/Name | Host origin | Species | Cell count  log_10_ (CFU/g) | % relative to 100-23 |
| --- | --- | --- | --- | --- |
| 100-23 | Rat | L. reuteri | 7.6 ± 0.4 | 100.0 |
| LR14 (pH52_RY) | Pheasant | L. balticus | 7.5 ± 0.2 | 79.4 |
| LR83 (BG-AF3-A) | Yellow-necked mouse | L. balticus | 8.0 ± 0.3 | 251.2 |
| LR95 (WF-MT5-A) | Field vole | L. agrestis | 5.7 ± 0.5 | 1.3 |
| LR103 (BG-MG3-A) | Bank vole | L. agrestis | 5.9 ± 1.0 | 2.0 |
| LR104 (BG-MG3-B) | Bank vole | L. agrestis | 4.8 ± 0.6 | 0.2 |
| LR75 (RRLNB_1_1) | Red ruffed lemur | L. albertensis | 8.0 ± 0.7 | 251.2 |
| LR76 (RRLNB_2_1) | Red ruffed lemur | L. albertensis | 8.1 ± 0.4 | 316.2 |
| LR91 (Lr3040) | Hamster | L. albertensis | 9.2 ± 0.1 | 3981.1 |
| LR116 (STM2_1) | Striped mouse | L. rudii | 7.1 ± 0.1 | 31.6 |
| LR117 (STM3_1) | Striped mouse | L. rudii | 7.3 ± 0.2 | 50.1 |
| LR96 (WF-MA1-A) | Common vole | L. fastidiosus | 5.3 ± 1.1 | 0.5 |
| LR97 (WF-MA3-B) | Common vole | L. fastidiosus | 5.6 ± 0.8 | 1.0 |
| LR100 (WF-MO7-1) | Root vole | L. fastidiosus | 5.4 ± 0.8 | 0.6 |

Cell numbers of strains of five *Limosilactobacillus* species colonized on the forestomach epithelium of germ-free mice were quantified by viable plate counts on MRS agar. Germ-free mice were inoculated by gavage with a single dose of different strains 3 days before sacrifice. The colonization of the forestomach epithelium is expressed as % relative to *L. reuteri* subsp. *rodentium* 100-23, for which the biofilm formation in mice is well-characterized. Cell densities of more than 6.9 log10 of CFU/g, 20% relative to *L. reuteri* subsp. *rodentium* 100-23 were considered as effective epithelial adhesion; cell densities of less than 10%, corresponding to less than 6.6 log10 of CFU/g, were considered as ineffective epithelial adhesion.

## Supplementary References

1. Page AJ, Cummins CA, Hunt M, Wong VK, Reuter S, Holden MT, et al. Roary: rapid large-scale prokaryote pan genome analysis. Bioinformatics. 2015;31:3691-3693.

2. Rambaut A, Drummond AJ, Xie D, Baele G, Suchard MA. Posterior summarization in Bayesian phylogenetics using Tracer 1.7. Syst Biol. 2018;67:901-904.

3. Drummond AJ, Rambaut A. BEAST: Bayesian evolutionary analysis by sampling trees. BMC Evol Biol. 2007;7:214.
